# Taking up the quest for novel molecular solar thermal systems: Pros and cons of storing energy with cubane and cubadiene

**DOI:** 10.3389/fchem.2023.1171848

**Published:** 2023-04-12

**Authors:** Cecilia Merino-Robledillo, Marco Marazzi

**Affiliations:** ^1^ Universidad de Alcalá, Departamento de Química Analítica, Química Física e Ingeniería Química, Alcalá de Henares, Madrid, Spain; ^2^ Universidad de Alcalá, Instituto de Investigación Química ‘‘Andrés M. del Río’’ (IQAR), Alcalá de Henares, Madrid, Spain

**Keywords:** molecular solar thermal systems, solar fuels, computational design, molecular photoswitches, multi-configurational quantum chemistry

## Abstract

Molecular solar thermal (MOST) systems are working their way as a possible technology to store solar light and release it when necessary. Such systems could, in principle, constitute a solution to the energy storage problem characteristic of solar cells and are conceived, at a first instance, as simple molecular photoswitches. Nevertheless, the optimization of their different required properties is presently limiting their technological scale up. From the chemical perspective, we need to design a novel MOST system based on unconventional photoswitches. Here, by applying multi-configurational quantum chemistry methods, we unravel the potentialities of *ad hoc*-designed molecular photoswitches, which aim to photoproduce cubane or cubadiene as high-energy isomers that can be thermally (or eventually catalytically) reverted to the initial structure, releasing their stored energy. Specifically, while cubane can be photoproduced *via* different paths depending on the reactant tricycle diene conformation, an undesired bicyclic by-product limits its application to MOST systems. An evolution of this starting design toward cubadiene formation is therefore proposed, avoiding conformational equilibria and by-products, considerably red shifting the absorption to reach the visible portion of the solar spectrum and maintaining an estimated storage density that is expected to overcome the current MOST reference system (norbornadiene/quadricyclane), although consistently increasing the photoisomerization energy barrier.

## 1 Introduction

The world’s main source of energy today is constituted by fossil fuels, although they are not a renewable source of energy. In addition to releasing CO_2_ into the atmosphere, this is one of the main causes of global warming (Rothenberg, 2023). For these reasons, in recent decades, constantly growing research studies are attempting to find alternative sources of energy with the objective to be cleaner, renewable, and sustainable (Banerjee et al., 2022). Among them, solar energy is one of the most affordable, since it does not require the production and management of waste as nuclear energy (Usman, 2022), and at the same time, it is freely available all over the Earth’s surface, with almost constant intensity, compared to wind (Roga, 2022).

Within solar energy technologies, different types of photovoltaics have been developed in the last 40–50 years with the goal of efficiently transforming solar energy into electrical energy that could then be used for any technological application (Xu and Qiao, 2011; Yum, 2013; Seo et al., 2016; Turan, 2016; Matsui, 2018; Schnappinger, 2018; Zhang, 2018; Blachowicz and Ehrmann, 2020; Huaulmé, 2020; Yu, 2021). Nevertheless, the main drawback of photovoltaics is their lack of capacity for storing the photogenerated electricity. More precisely, energy storage is possible only when coupling photovoltaics to high-volume and expensive lithium batteries (Üçtuğ and Azapagic, 2018). Hence, one of the current main challenges is no longer the transformation of solar energy but its storage. In this respect, solar technology shares its fate with hydrogen technology since consistent improvements have been made on the side of molecular dihydrogen production efficiency (Lucarini, 2020; Lucarini, 2021; Meng, 2023; Wang, 2023; Zhang, 2023), but its large-scale storage is still under debate (Andersson and Grönkvist, 2019; Elberry, 2021).

**FIGURE 1:**
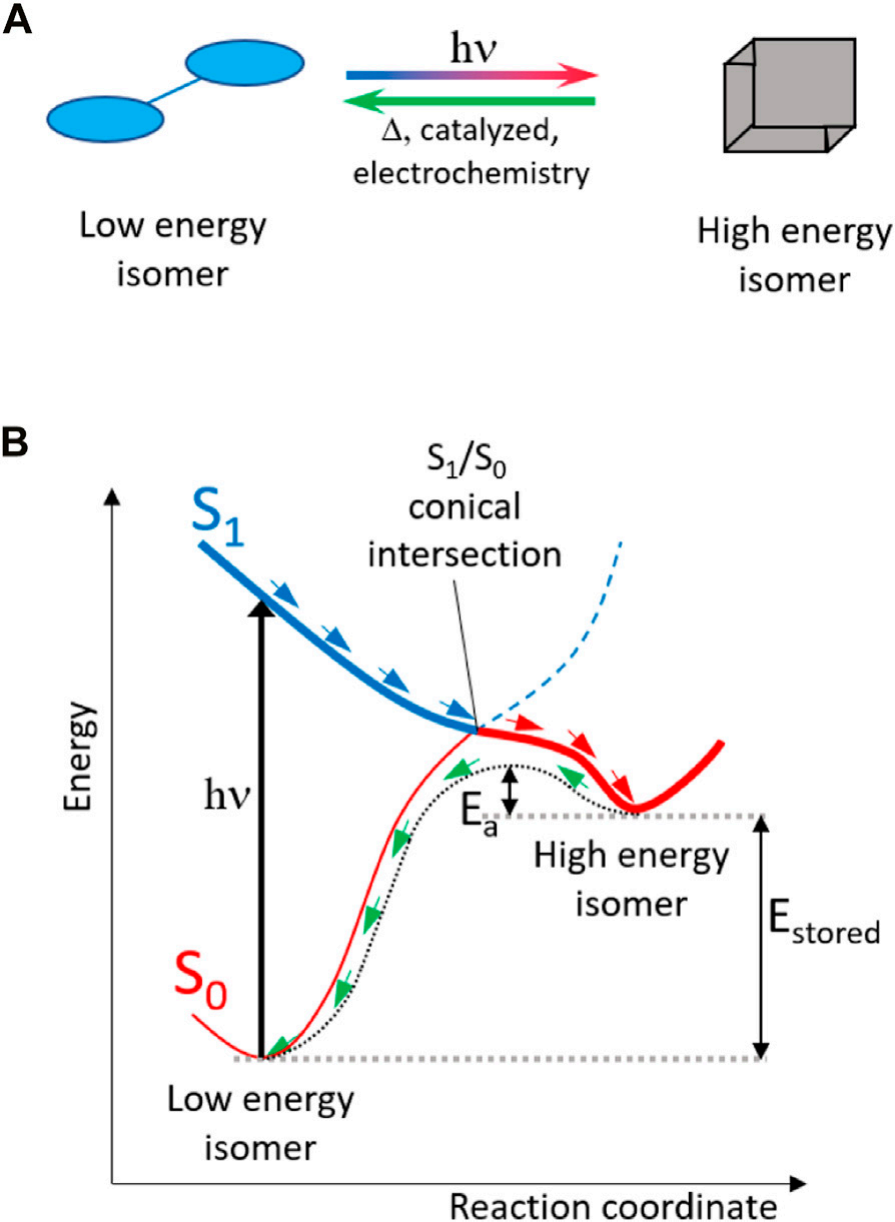
Functioning concept of a molecular solar thermal (MOST) system: **(A)** solar irradiation (hν) can convert a low-energy isomer into a stable high-energy isomer. **(B)** The stored energy (E_stored_) can be released on demand by overcoming the ground-state activation energy (E_a_) by applying heat, a proper catalyst, or through electrochemistry.

Concerning solar energy, few solutions were indicated, one of them being the development of more performant and/or cheaper batteries, and the other one relying on the concept of the molecular solar thermal (MOST) system (Lennartson et al., 2015; Sun et al., 2019; Xu and Wang, 2022) a molecule could be designed as a low-energy isomer that can be activated by solar light and transformed into a high-energy isomer (Figure 1). The difference in energy between the two isomers constitutes the stored energy (E_stored_). Apart from high photon absorption intensity and efficiency in the photoisomerization process, the latter usually depending on the topology of the conical intersection between excited (S_1_) and ground (S_0_) electronic states, other criteria need to be fulfilled to obtain a suitable MOST system.

- The high-energy isomer has to be photochemically stable at room temperature, meaning that it cannot be reverted back to the low-energy isomer by the same sunlight.
- The ground state energy barrier (i.e., the activation energy, E_a_) to release the stored energy has to be high enough to guarantee chemical stability of the high-energy isomer, and at the same time, it should be conveniently lowered by the addition of a proper catalyst (Moth-Poulsen, 2012; Lorenz et al., 2021) or by electrochemical methods (Franz, 2022) to release E_stored_ at demand.
- To be of technological interest, the storage density (D_storage_ = ∆H_storage_/M, where ∆H_storage_ is the difference in enthalpy between high- and low-energy isomers and M is the molecular weight) needs to be maximized, therefore requiring a small-sized molecule capable of the highest possible photo-induced enthalpy increment.
- For device purposes, the overall MOST system (i.e., both isomers) should be liquid to ensure a physical separation of high- and low-energy isomers.

Therefore, it can be realized that, although simple in its chemical concept, the optimization of a MOST system is far from being a trivial task.

Historically, the MOST concept was developed in 1909 through the photodimerization of anthracene molecules specifically planned to store solar energy (Weigert, 1909). Much later, several MOST systems were proposed starting from the ‘80s, including different mechanisms, such as photocyclization of the dihydroazulene/vinylheptafulvene system (Cacciarini, 2015; Wang, 2017; Mogensen, 2019; Nielsen, 2020), *cis*/*trans* photoisomerization of stilbene and azobenzene derivatives (Masutani et al., 2014; Dong, 2018; Hu, 2019; Sun et al., 2019; Wang, Losantos, et al., 2019; Zhang et al., 2022), photoisomerization of bimetallic complexes (Zhang, 2013), and [2 + 2] photocycloaddition of the norbornadiene/quadricyclane system (Kuisma, 2016; Jevric et al., 2018). Some systems, although initially discarded for their poor properties, were recently taken back into account, in light of methodological developments concerning mechanochemistry applied to azobenzene (Marazzi and Garcia-Iriepa, 2019; Nucci et al., 2019), electrochemistry (Franz, 2022), and novel synthetic routes to derivatize the norbornadiene/quadricyclane system (Orrego-Hernández et al., 2020). This latter scientific advancement consolidated, in these last years, the norbornadiene/quadricyclane system as the most promising for MOST applications (Bren’, 1991; Jorner, 2017; Elholm, 2022), mainly due to the considerably higher E_stored_ compared to the rest of the proposed systems and, at the same time, its relatively low molecular weight, hence maximizing D_storage_ (see the previous section for its definition). The main drawback remains its absorption in the UV region (*λ* < 267 nm), which can be only partially solved by proper substitution patterns: a *push*–*pull* effect can be efficiently obtained by introducing a -CN group as an electron acceptor and a -Ph-OCH_3_ group as an electron donor, reaching only partially the desired visible absorption [*λ* < 558 nm (Wang, et al., 2019)] and mainly compromising the D_storage_ value due to a consistent increase in molecular weight. Moreover, norbornadiene is plagued by severe solubility issues, making it difficult to be prepared as a liquid (Maruyama et al., 1985; Quant, 2016).

Here, we propose an alternative to the well-known norbornadiene/quadricyclane system and to all previously studied systems: tricyclooctadiene (TOD) and, especially, tricyclooctatetraene (TOT), with the goal of photoactivating them to produce all-carbon cubane (CUB) or cubadiene (CUD), respectively. Organic cubanes are known in the literature to be potential explosives (Engelke and Stine, 1990; Eaton et al., 2000; Qin, 2015); hence, they are expected to store a large amount of energy while keeping the atom economy to a reduced number of organic weighted atoms (eight carbon atoms). Until now, only the TOD/CUB system was theoretically proposed for MOST purposes, showing some of its drawbacks (mainly, the possible formation of by-products). The TOT/CUD system, on the other hand, was never proposed. Through multi-configurational quantum chemistry methods, we will show the electronic absorption properties, photochemical, and ground-state mechanistic paths of both TOD/CUB and TOT/CUD systems, highlighting their pros and cons in the quest for novel MOST systems.

## 2 Methodology

### 2.1 Design strategy

**FIGURE 2:**
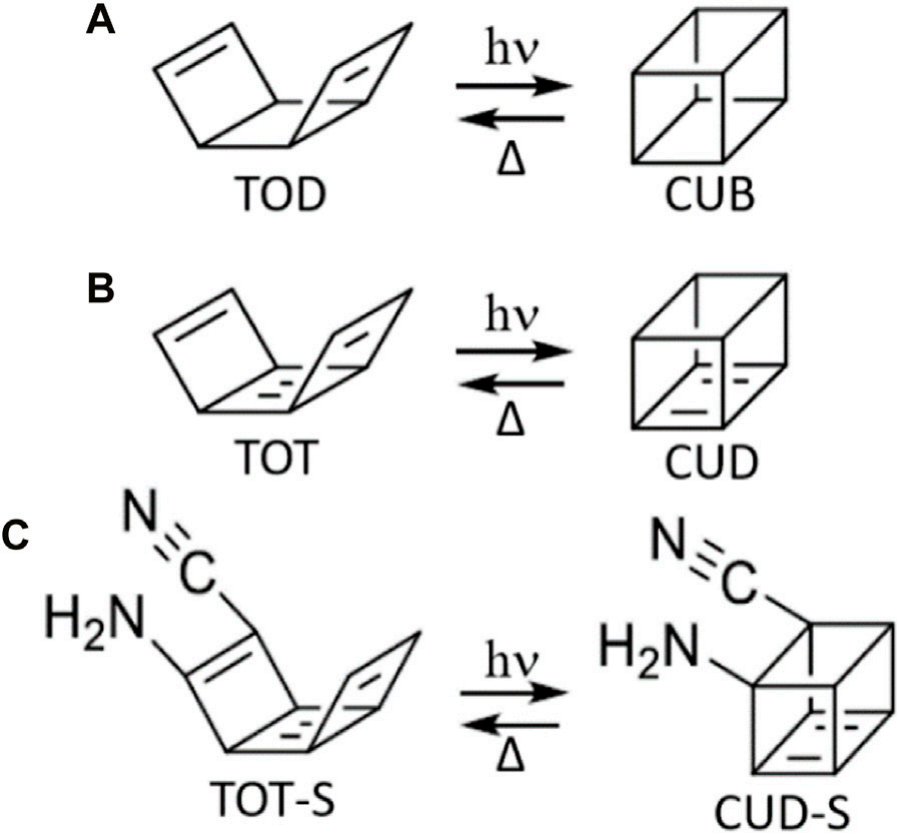
**(A)** TOD/CUB system, leading to the photochemical formation of cubane; **(B)** TOT/CUD system, leading to the photochemical formation of cubadiene, and **(C)** its substituted derivative TOT-S/CUD-S. To serve as molecular solar thermal systems, the photoproduct needs to be a photochemically stable high-energy isomer and should be capable of thermal reversion to its initial low-energy isomer.

The photochemical step of the proposed TOD/CUB system is based on the same [2 + 2] photocycloaddition (photo-induced Diels–Alder) reaction characteristic of the norbornadiene/quadricyclane system. Nevertheless, in this case, the objective was to photoproduce a higher-energy isomer due to its higher steric strain, as also suggested by previous theoretical and experimental studies on similar compounds that indicate all-carbon CUB derivatives as possible explosives due to their capacity of storing high amounts of energy within their molecular structures. Merging these two properties, i.e., the photoproduction of an all-carbon-based cubane by irradiating a diene capable of [2 + 2] photocycloaddition, we have proposed the TOD/CUB system (Figure 2A), where TOD stands for tricyclo [4,2,0,0^2,5^]octa-3,7-diene.

Additional possible ground- or excited-state pathways, eventually leading to different by-products, were not considered on the basis of the recent application of machine learning-based algorithms and extensive non-adiabatic molecular dynamics to cubane-based systems (Li and Lopez, 2020; Li, 2021).

As explained in the *Results and discussion* section, the undesired presence of a by-product within the TOD→CUB mechanism (indicated as BOT; bicyclo [4,2,0]octa-2,4,7-triene) led us to the design of the derived TOT/CUD system, where TOT stands for tricycloocta-1,3,5,7-tetraene and CUD for cubadiene; by including a central butadiene moiety (Figure 2B), we avoid ring-opening issues experienced by the TOD/CUB system; thus, no by-product is expected. Moreover, the inclusion of two additional π bonds considerably enlarges the conjugation length, allowing to reach photon absorption within the visible portion of the spectrum. A substitution pattern was considered for the successful TOT candidate, resulting in TOT-S, characterized by the inclusion of a -NH_2_ donor group and a -CN acceptor group at both ends of a C=C bond involved in photocyclization (Figure 2C). In this way, a *push*–*pull* effect was introduced in the TOT chromophore with the goal of partially changing the S_1_ state character by introducing a certain degree of charge transfer character, responsible for a bathochromic shift in absorption energy. Moreover, the reduced weight of the two selected substituents is expected not considerably to decrease its storage density.

### 2.2 Computational details

Due to the diradical nature of electronic ground transition states and the multi-configurational nature of electronic excited states, all calculations were performed at the CASPT2//CASSCF level, that is, geometry optimization by complete active space self-consistent field (CASSCF) theory (Olsen, 2011), followed by energy correction on top of the CASSCF calculation through second-order perturbation theory (CASPT2) (Andersson et al., 1992). This strategy was proven to be successful for the quantitative description of several photoisomerization processes (Marazzi and Garcia-Iriepa, 2019) based on [2 + 2] photocycloaddition (Runčevski, 2014). The 6-31G (d,p) basis set was used including a “double-zeta” approach for the valence orbitals and polarization functions (d,p) to better describe formation and rupture of chemical bonds.

The selected complete active space (CAS) is composed of six electrons and six orbitals for TOD, while eight electrons and eight orbitals were chosen for TOT. More in detail, such CASs allow including, in both cases, the full π system, if looking at the low-energy respective isomer and an additional *σ*-bond to correctly describe the eventual BOT formation. These result in 1 σ/σ* and 2 π/π* orbitals for TOD and 1 σ/σ* and 3 π/π* orbitals for TOT. The substituent effect was evaluated for TOT, concerning both photophysics (electronic absorption spectrum) and photochemistry (chemical reactivity due to photon absorption), as shown in Figure 2C. In this case (TOT-S), the selected CAS was increased until it included 12 electrons and 12 molecular orbitals. Moreover, the effect of the basis set was evaluated by considering the atomic natural orbitals (ANO-S and ANO-L). The results, shown in Supplementary Table S1, point toward a limited bathochromic shift (<10 kcal·mol^-1^) while overall maintaining the electronic description of ground and excited states. Therefore, the results shown for TOT-S were calculated with a CAS (8,8) as for TOT due to the delocalization of the molecular orbitals including TOT-S donor and acceptor groups.

The CASSCF calculations included two electronic states (S_0_ and S_1_), as it was found that S_2_ is much higher in energy. Equal weights were given to the two states in a state-average fashion (de Meras et al., 1990), resulting in a SA(2)-CASSCF type of calculation. Concerning CASPT2, three electronic states were included by a multi-state formalism (MS(3)-CASPT2) (Finley, 1998; Pulay, 2011). No IPEA correction was included, as it was found to be a reliable setting for many organic-based chromophores and not even including any preconceived bias (Zobel et al., 2017). Moreover, a 0.2 imaginary shift was considered in order to avoid state-order flipping (Forsberg and Malmqvist, 1997).

All calculations were performed in the gas phase, i.e., not including any solvent effect since, in principle, an optimal MOST system should be a pure liquid composed only of solar-thermal active molecules in order to maximize the storage density. We should note that in comparable MOST systems, as the norbornadiene/quadricyclane isomerization couple, a pure liquid cannot be attained and solvents need to be added; in any case, apolar organic solvents do not make valuable contributions to the solvatochromic effect (Orrego-Hernández et al., 2020). Moreover, due to the lack of experimental data for TOD, TOT, and TOT-S, several experimental solubility issues compromise a straightforward selection of the solvent (or mixture of solvents) that could be potentially modeled in this study.

Minimum energy paths were calculated for both excited and ground states by connecting featuring geometries as energy minima, intermediates, transition states, and conical intersections among potential energy surfaces. Where necessary, linear interpolations among an initial and a final structure were used, in order to initially calculate the minimum energy path. All CASSCF calculations were performed using Gaussian 16 software (Frisch, 2016), while CASPT2 energy corrections on top of CASSCF-optimized structures were performed using the OpenMolcas suite of programs (Fernández Galván et al., 2019).

The simulation of the absorption spectra, including their shape (Figure 8), was performed for TOD (*syn* and *anti*conformers), TOT, and TOT-S as follows: the oscillator strengths corresponding to the first two vertical excitation energies (S_0_→S_1,2_) were computed at the CASPT2 level using OpenMolcas software, corresponding to the maximum intensities of each respective band. Then, to calculate the spectral broadening, a Gaussian function was assigned to each vertical transition with a full width at half maximum (FWHM) of 0.2 eV. This specific value was proven to be valid in reproducing experimental spectra of different families of organic chromophores (Gattuso, 2016; Turan, 2016; Gattuso et al., 2017; Marazzi, 2018). Finally, all Gaussians were convoluted for each species. All details are given in the Supplementary Material.

## 3 Results and discussion

### 3.1 The TOD/CUB system

**FIGURE 3:**
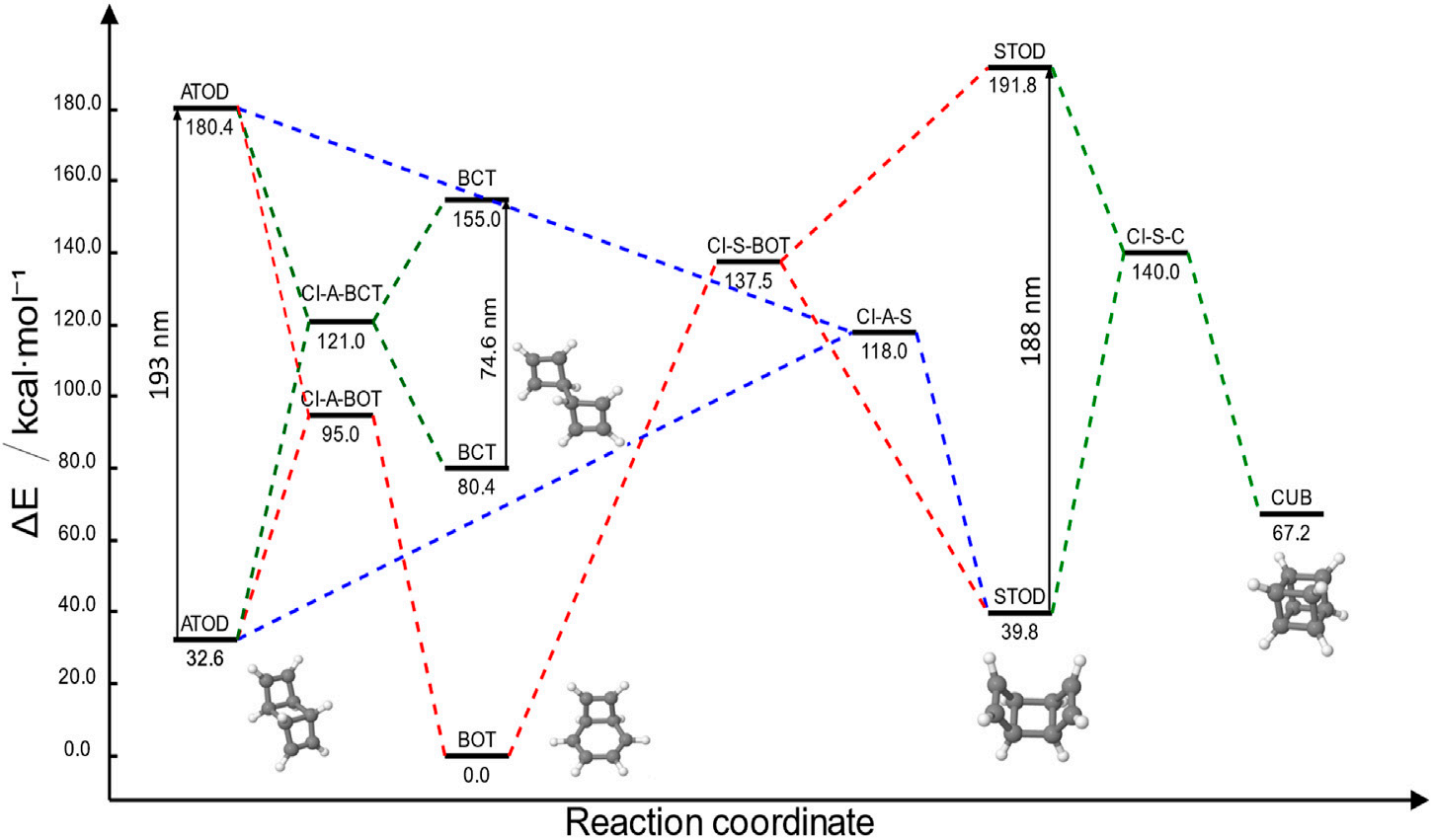
Summary of the TOD/CUB photochemistry in terms of CASPT2 energy referred to the most stable structure (BOT). All ground-state geometries corresponding to different species involved are shown. Vertical arrows correspond to S_0_→S_1_ photon absorption. All S_1_/S_0_ conical intersections (CI) are shown. Different colors correspond to the possible photochemical pathways, including photoproduct and reactant recover: ATOD→STOD (dotted blue), ATOD→BCT (dotted dark green), ATOD→BOT and STOD→BOT (dotted red), and STOD→CUB (dotted olive).

The TOD/CUB desired reactivity is shown in Figure 2A. Nevertheless, TOD has two possible conformations: *syn* and *anti*, from here on STOD and ATOD, respectively. On the one hand, STOD has both cyclobutene lateral moieties pointing toward the same direction with respect to the middle cyclobutane moiety. On the other hand, ATOD is 7.2 kcal·mol^-1^ more stable than STOD, but since it is characterized by two cyclobutene lateral moieties pointing in opposite directions with respect to the middle cyclobutane moiety, it does not keep the two C=C moieties near enough for an eventual [2 + 2] photocyclization. Therefore, the reactivity of Figure 2A is only possible once STOD is formed. This gives rise to a complex photochemical scenario, summarized in Figure 3.

Specifically, STOD can be photoproduced by directly irradiating ATOD through a conical intersection (CI-A-S, dotted blue path). Once STOD is generated, a second photon with almost the same energy can produce the desired CUB through the CI-S-C conical intersection (dotted green path). As expected, both CIs could also lead to internal conversion, generating back the initial respective reactant. Nevertheless, two main drawbacks can be observed for the MOST application:

1) The required photon energy for both photoreactions is completely within the UV range, a problem common to many other compounds that were initially explored as possible MOST systems, including the known norbornadiene/quadricyclane system and, in principle, improvable by convenient substitution patterns, aiming at red shifting the absorption energy.
2) The ATOD $\overset{h\nu }{\to }$ STOD $\overset{h\nu }{\to }$ CUB path is not the only one possible. Indeed, STOD excitation to the S_1_ state can also open a second channel that, through the CI-S-BOT conical intersection, could form a stable by-product, bicyclo [4,2,0]octa-2,4,7-triene (BOT), as a result of C–C bond breaking and formation of a six-carbon membered ring. Additionally, BOT could also be formed as a second possible channel of ATOD S_1_ excitation, through the CI-A-BOT conical intersection.

BOT formation as the main inconvenience of the TOD/CUB system was already proposed by another theoretical study (Li and Lopez, 2020), although in that case, only the *syn* TOD conformation was considered, therefore narrowing the generality of the conclusions on the eventual use of TOD as a MOST system.

For the sake of completeness, it should be mentioned that ATOD S_1_ excitation also has, in principle, a third possible deactivation channel, forming a metastable diradical (a bicyclic tetraene: BCT) through the CI-A-BCT conical intersection. Although BCT can absorb light almost in the visible range (383 nm), the only possible evolution is internal conversion to form back ATOD through the same CI-A-BCT conical intersection, hence not constituting an interesting pathway for our purposes.

Concerning E_stored_, the TOD/CUB system can store 34.6 kcal·mol^-1^, considering its stable ATOD structure. This value overcomes the 22.9 kcal·mol^-1^ found for the unsubstituted norbornadiene/quadricyclane reference (Nucci et al., 2019). In terms of D_storage_ too, the 1.39 kJ·g^-1^ of TOD/CUB would result in a consistent improvement compared to the bare norbornadiene/quadricyclane system (1.04 kJ·g^-1^). Nevertheless, since the BOT by-product can be formed by either irradiating ATOD or STOD, its use as a MOST is definitely hampered. In Figure 3, we focused our attention on excitation energies and ground-state relative energies. Nevertheless, energy barriers for all the aforementioned pathways should be discussed to understand their feasibility. This is shown in the following Section 3.1.1 for ATOD, and Section 3.1.2 for STOD.

#### 3.1.1 ATOD photo-reactivity

**FIGURE 4:**
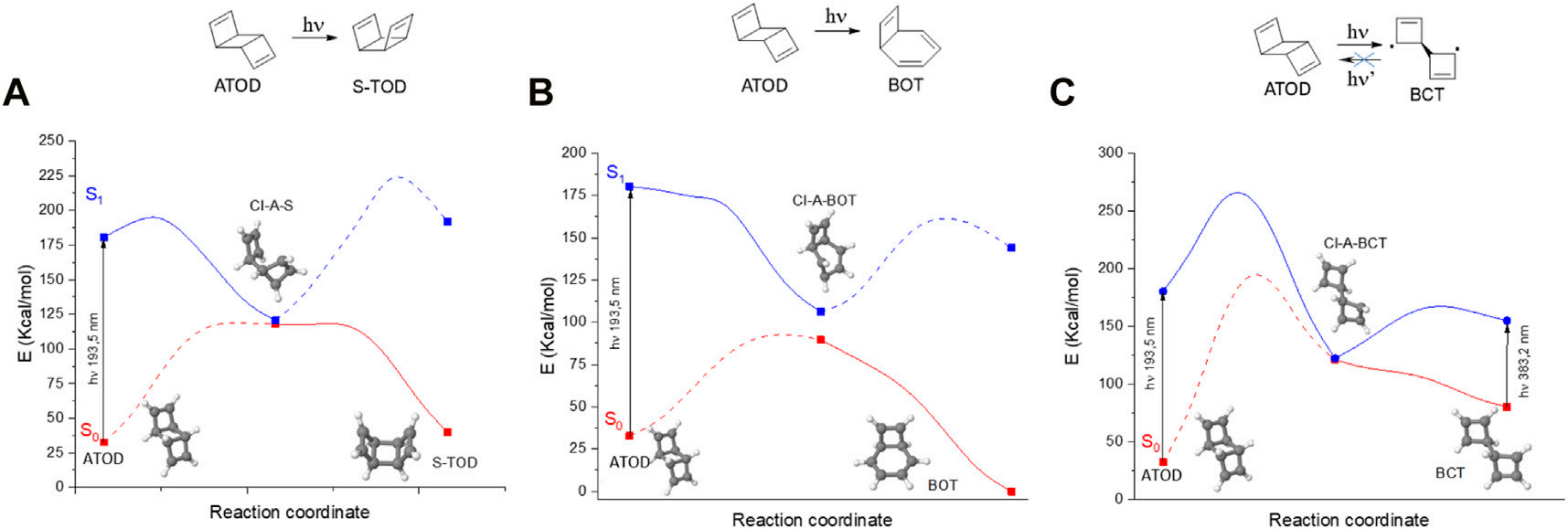
Different CASSCF photochemical pathways initiated by ATOD S_0_→S_1_ photon absorption: **(A)** ATOD→STOD, **(B)** ATOD→BOT, **(C)** ATOD→BCT, and their respective internal conversion. Energy graphs are shown in adiabatic fashion (S_0_: red and S_1_: blue) with the continuous lines corresponding to the populated potential energy surfaces necessary to reach the respective photoproduct. Geometrical structures of S_0_ minima and S_1_/S_0_ conical intersections are also shown.

As we have discussed in the previous section, three deactivation channels are, in principle, possible when irradiating ATOD into the S_1_ excited state: photoproduction of STOD (Figure 4A), BOT (Figure 4B), and BCT (Figure 4C).

Comparing the S_1_ deactivation pathways for the three cases, it can be concluded that the most probable channel is constituted by BOT photoproduction, since it is the only energy barrierless process. On the other hand, STOD photoisomerization requires a *ca.* 12 kcal·mol^-1^ barrier, and BCT formation requires a much higher 85 kcal·mol^-1^ barrier. Therefore, since ATOD is the most stable TOD isomer, and BOT photoproduction is unavoidable, this is confirmed as a firm limit of the TOD/CUB system for MOST applications.

#### 3.1.2 STOD photo-reactivity

**FIGURE 5:**
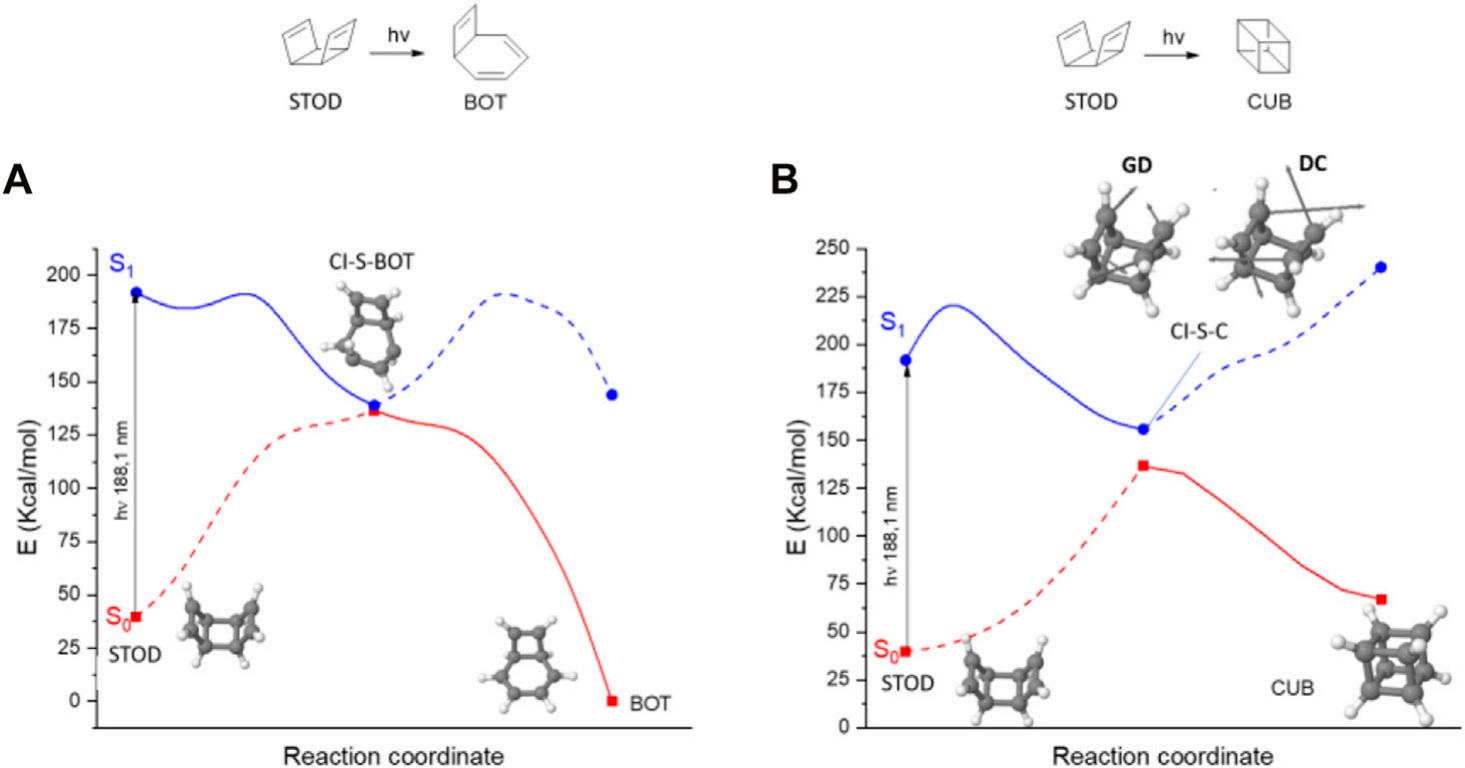
Different CASSCF photochemical pathways initiated by STOD S_0_→S_1_ photon absorption: **(A)** STOD→BOT and **(B)** STOD→CUB. The energy graph is shown in adiabatic fashion (S_0_: red and S_1_: blue) with the continuous lines corresponding to the populated potential energy surfaces necessary to reach the respective photoproduct. Geometrical structures of S_0_ minima and S_1_/S_0_ conical intersections are shown, including in the case of CI-S-C the non-adiabatic coupling vectors: gradient difference (GD) and derivative coupling (DC) vectors.

Even if STOD could be eventually populated by thermal chemistry (see next section), once excited to S_1_, STOD photodeactivation is once again biased in favor of BOT formation (energy barrier of 11 kcal·mol^-1^) compared to the desired CUB formation (29 kcal·mol^-1^), as shown in Figure 5. Nevertheless, it should be noted from the CI-S-C conical intersection structure and especially from the direction that its non-adiabatic coupling vectors indicate that a photochemical Diels–Alder reaction is expected to produce CUB, as usual for [2 + 2] photocycloadditions (inset of Figure 5B).

#### 3.1.3 Energy release from CUB

**FIGURE 6:**
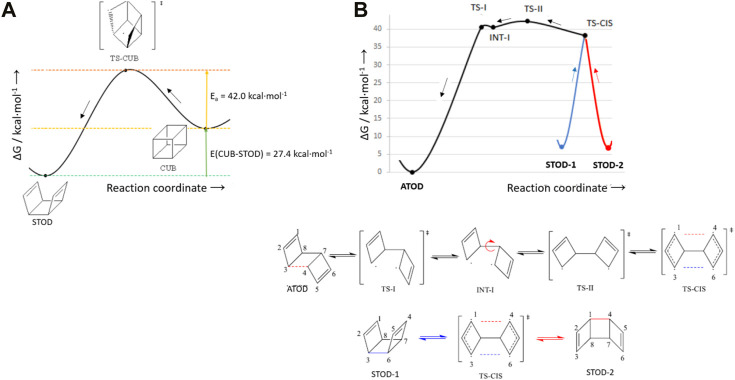
Two steps to release, on the ground state, the energy stored by CUB: **(A)** CUB→STOD through the transition state TS-CUB and **(B)** STOD→ATOD through a diradical intermediate INT-I.

Although we have proved that, by irradiating TOD, the desired formation of CUB is partially hampered by the stabilization of BOT as a by-product, we have also investigated the following step of a MOST system, i.e., energy release from the high-energy isomer CUB. Indeed, in principle, CUB could also be formed by alternative photochemical strategies with respect to the one that we show, possibly sharing a [2 + 2] photocycloaddition mechanism. In this specific case, energy release involves two steps: CUB→STOD first and STOD→ATOD later. More in detail, the first CUB→STOD step is the essential one, since it is responsible for the release of most of the stored energy (27.4 kcal·mol^-1^), and once formed, STOD could absorb light by starting a new MOST cycle. Specifically, this first step requires overcoming a barrier of 42.0 kcal·mol^-1^ involving the rupture of one of the twelve equivalent C–C *s*-bonds present in CUB, thus formally generating a transition state characterized by a diradical structure (TS-CUB in Figure 6A).

Nevertheless, since ATOD is lower in energy than STOD by 7.2 kcal·mol^-1^, the population of ATOD is thermodynamically largely favored, and therefore, STOD light absorption is in competition with STOD→ATOD ground-state isomeric conversion. Moreover, due to its intrinsic symmetry, four chemically equivalent STOD isomers can be interconverted into each other, with a thermal barrier of *ca.* 30 kcal·mol^-1^ (Supplementary Figure S1). In Figure 6B, we show how the ATOD compound can be restored, starting from two equivalent STOD-1 and STOD-2 structures and connected by the TS-CIS transition state. From there, a metastable intermediate of a diradical character (INT-I) was located approximately 40 kcal·mol^-1^ higher in energy than ATOD.

All in all, we can conclude that, among the two release steps, the CUB→STOD one requires more energy (42.0 kcal·mol^-1^) than the STOD→ATOD step (35.1 kcal·mol^-1^), and therefore, STOD could safely serve as a stable high-energy isomer. Interestingly, if a proper catalyst could be found to catalyze the CUB→STOD step [in principle, requiring a retro Diels–Alder reaction, e.g., the quadricyclane→norbornadiene reaction catalyzed by specific metal-based complexes (Bauer, 2018; Jevric, 2018; Wang et al., 2019; Luchs et al., 2020)], then the formed STOD preferentially keeps its *syn* conformation, since interconversion among STOD-equivalent isomers is energetically more feasible than adopting the initial *anti* conformation.

### 3.2 The TOT/CUD system

#### 3.2.1 Cubadiene photochemical production

As discussed in Section 3.1, the TOD/CUB system is largely unsuitable to store solar energy, mainly because of TOD C–C *σ*-bond breaking that results, during the irradiation step, in the formation of BOT, a bicyclic triene stabilized by a six-carbon diene ring. Nevertheless, the encouraging E_stored_ and D_storage_ values compared to the norbornadiene/quadricyclane reference made us think about how to circumvent such problem, finding a possible solution for the TOT/CUD system. The TOT low-energy isomer bears the same tricyclic structure as TOD, but the central four-carbon ring is now a cyclobutadiene moiety, instead of a cyclobutane moiety. This design modification brings several advantages:

1) Due to the conjugation of the central ring, no C–C *s*-bond breaking is expected, neither photochemically nor thermally, thus avoiding the formation of stable bicyclic by-products.
2) The overall π conjugation is now extended, passing from two separated π bonds to four conjugated π bonds, hence expecting a consistent bathochromic shift in the TOT absorption spectrum, compared to TOD (see Section 3.3).
3) Although, in principle, a *syn* and an *anti*conformer could also be sketched for TOT, and their thermal equilibrium cannot take place since it is not feasible for a fully π-conjugated molecule to break a C–C bond (now stronger than a simple *s*-bond, due to partial π character), rotate, and isomerize, as described in Section 3.1.3 and depicted in Figure 6B. We should therefore consider that, depending on the synthetic route, either the *syn* or the *anti* conformer can be obtained, and we are interested in *syn* TOT as our low-energy isomer, having both lateral C=C moieties in a convenient spatial arrangement to undergo the desired [2 + 2] photocycloaddition and form cubadiene.

**FIGURE 7:**
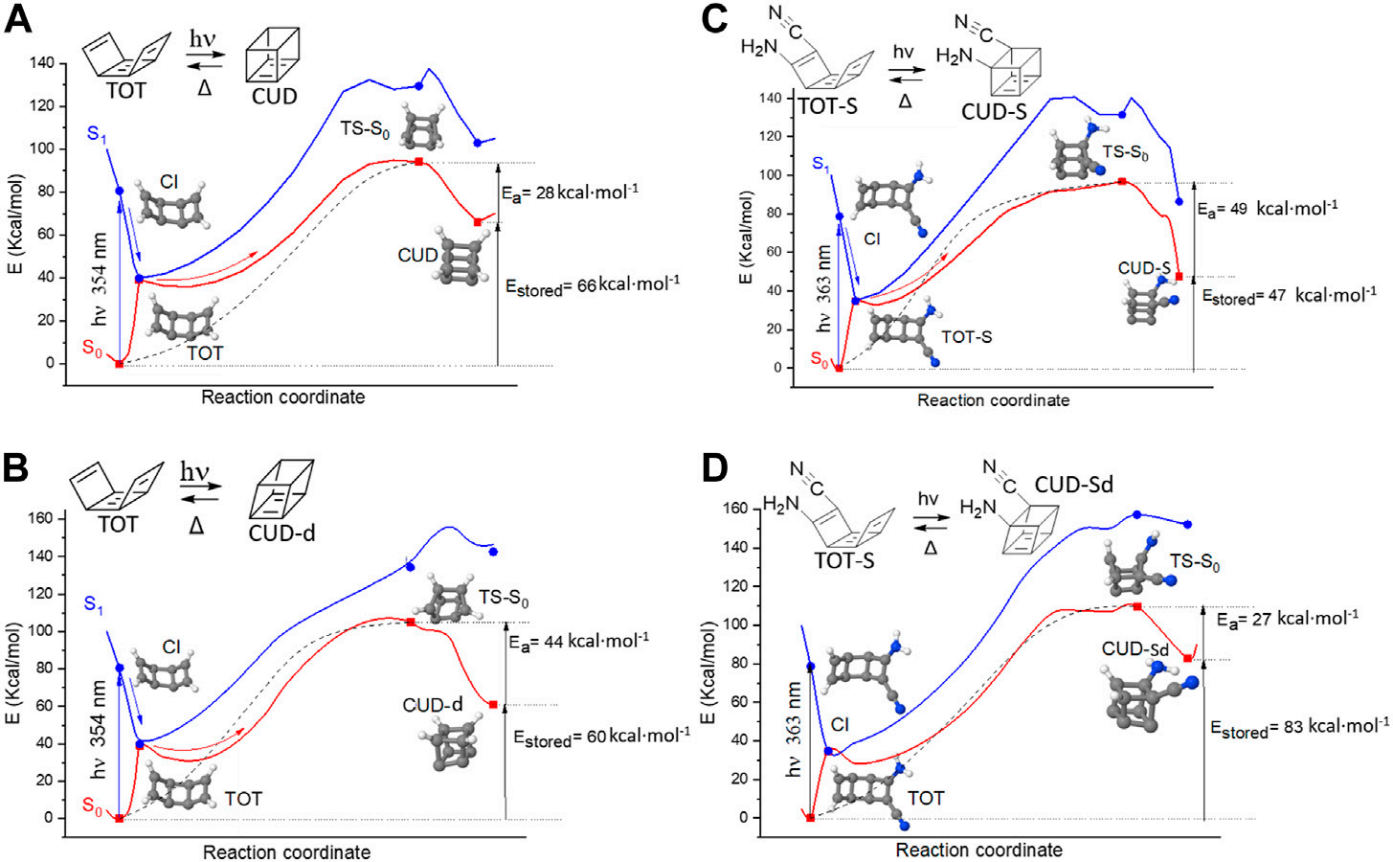
Photoproduction of **(A)** CUD and **(B)** distorted CUD from TOT and photoproduction of **(C)** the substituted CUD and **(D)** substituted and distorted CUD from the substituted TOT. The ground-state reversion path to TOT **(A,B)** and TOT-S **(C, D)** is also depicted by a dotted line. All minimum energy paths show CASSCF energetics and the structures of minima, conical intersections (CI), and S_0_ transition states (TS).

Moreover, as shown in Figures 7A, B, two different cubadiene structures can be obtained, a symmetric (CUD) and a distorted (CUD-d) one, with the symmetric photoproduct making it possible to store slightly more energy than the distorted one. The drawback of the process is constituted by a S_1_/S_0_ conical intersection that, in both cases, does lead to CUD or CUD-d only if overcoming a relatively large ground-state energy barrier of *ca.* 50–60 kcal·mol^-1^ at both CASSCF and (see Supplementary Figure S2) CASPT2 levels. Even if the ground state is populated with a consistent excess energy given by S_0_→S_1_ photon absorption, 10–20 extra kcal·mol^-1^ would be anyway necessary, being, in principle, at disposition through a slight hypsochromic absorption and/or taking advantage of the expected high temperature of the MOST liquid when exposed under the Sun (i.e., it is expected that high vibrational levels should be propagated in the ground state). In any case, this barrier clearly constitutes the main limit to the whole process.

The substituent effect was considered for TOT by following the same norbornadiene design principle of adding a donor group (-NH_2_) and an acceptor group (-CN) to the same C=C lateral bond, thus inducing a *push*–*pull* effect. This results in the TOT-S/CUD-S system, where S stands for substituted. As shown in Figures 7C, D (CASSCF level) and in Supplementary Figure S3 (CASPT2 level), the same mechanism found for the unsubstituted TOT/CUD system also applies here; both cubadiene isomers can be formed, but at the expense of a ground-state energy barrier. As a positive effect, light absorption by TOT-S is expected to be red shifted, as we show and rationalize in the next section.

Concerning E_stored_, we should note that, among the symmetric and the distorted cubadiene, the system will preferentially evolve toward the photoproduct involving the lower-energy S_0_ barrier. Although near in energy, this reasoning would favor the formation of CUD over the distorted CUD-d, when TOT is unsubstituted (E_stored_ = 51 kcal·mol^-1^ at the CASPT2 level), while CUD-Sd would be preferred over CUD-S, when TOT is substituted (E_stored_ = 32 kcal·mol^-1^ at the CASPT2 level). Reminding the value found for the ATOD/CUB system (34.6 kcal·mol^-1^ at the CASPT2 level), this means that E_stored_ (ATOT/CUD) >> E_stored_ (TOD/CUB) ≈ E_stored_ (TOT-S/CUD-Sd). In terms of D_storage_, the superiority of the TOT/CUD system is guaranteed by the fact that the molecular weight is 4 g·mol^-1^ lower than the TOD/CUB system, due to the absence of the four hydrogen atoms connected to the middle TOT ring. On the other hand, although the TOT-S/CUD-Sd is expected to be the system offering the best optical properties, its increased molecular weight (due to the presence of donor and acceptor groups) would result in a decrease in the storage density, compared to its unsubstituted analogs. E_stored_ and D_storage_ values are presented in Table 1.

**TABLE 1:** Storage energy (E_stored_) and storage density (D_storage_) for different molecular compounds evaluated in this study as potential MOST systems, compared to unsubstituted norbornadiene/quadricyclane (NBD/QC).

Molecular system	E_stored_/kcal·mol^-1^	D_storage_/kJ·g^-1^
NBD/QC	22.9	1.04
ATOD/CUB	34.6	1.39
STOD/CUB	27.4	1.10
TOT/CUD	51.0	2.13
TOT/CUD-d	29.0	1.21
TOT-S/CUD-S	52.0	1.55
TOT-S/CUD-Sd	32.0	1.34

#### 3.2.2 Energy release from cubadiene

Mechanistically, Figure 7 shows that the same S_0_ transition state is involved when “photo-charging” the MOST system and when releasing the stored energy (on the ground state), constituting a particularity never proposed until now for a molecular solar fuel. In terms of photoproduct stability, we note that, at the quantitative CASPT2 level, the distorted cubadienes (both CUD-d and CUD-Sd) offer the highest energy barriers to release the stored energy (50 and 26 kcal·mol^-1^, respectively) although, as aforementioned, they store less energy (29 and 32 kcal·mol^-1^, respectively) compared to the symmetric cubadienes, CUD and CUD-S, which show slightly lower energy barriers of 24 and 23 kcal·mol^-1^, respectively, but allowing to store 51 and 52 kcal·mol^-1^, respectively (Supplementary Figures S2 and S3).

### 3.3 Absorption spectra

The main unresolved issue of the most established molecular solar fuel, the norbornadiene/quadricyclane system, is the absorption of photons within the solar spectral range. Indeed, as explained in the *Introduction*, the absorption of the unsubstituted norbornadiene/quadricyclane system lies completely in the UV range, and although several synthetic strategies were developed in the last 30 years to red shift its absorption, it was possible to include only a part of the visible window, centered in the blue with the tail covering the green window (Wang, et al., 2019).

**FIGURE 8:**
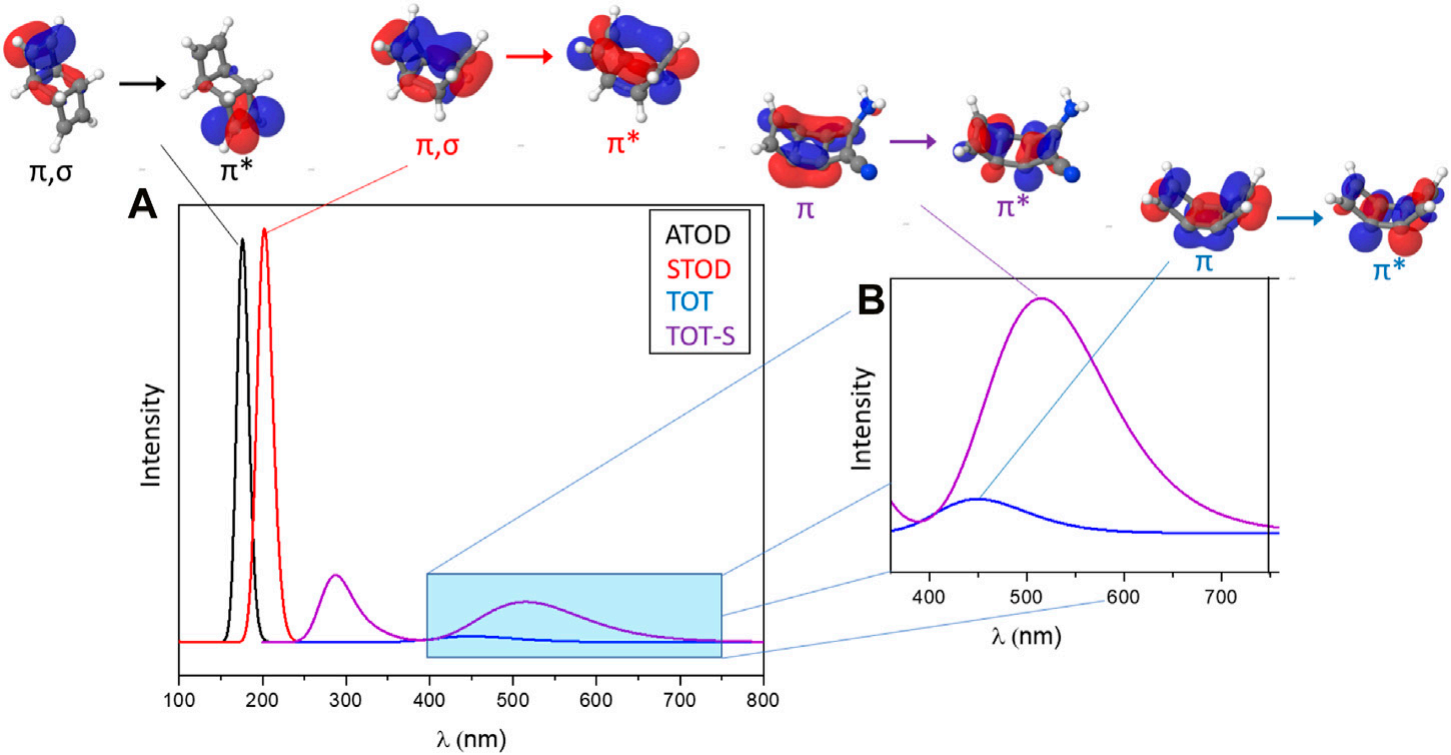
**(A)** Simulated absorption spectra in the UV-visible spectral range, including **(B)** a zoom covering only the visible window, of interest for MOST applications. Each S_0_→S_1_ band is assigned to the respective couple of occupied and virtual orbitals.

The photoproduction of cubanes by the TOD/CUB system shows (Figure 8, black and red bands) the same exact problem, since the S_0_→S_1_ vertical transition of both *anti* and *syn* TOD conformations fall in the UV spectral range (*λ* < 250 nm). Nevertheless, when considering the more promising photoproduction of cubadienes (CUD), the absorption of the unsubstituted TOT is, although much weaker in intensity, centered around 450 nm, which is already within the visible region (Figure 8, blue band). We note that, although a bright intensity would be surely beneficial for the efficiency of the photoprocess, a low absorption intensity does not compromise the photoisomerization process and, thus, its potential use as a MOST component [see, e.g., azobenzene (Bandara and Burdette, 2012; Zapata, 2014; Aleotti, 2020)]. Substituted TOT (TOT-S) shows, as expected due to the introduced *push*–*pull* effect, an even larger bathochromic shift that makes it, in principle, suitable to absorb light almost within the full visible spectrum (400–700 nm), together with an increased intensity compared to TOT (Figure 8, violet band).

When looking at the nature of the vertical transitions, we can conclude, based on molecular orbitals analysis, that ^1^(π, π*) transitions of ATOD and STOD are partially contaminated by σ-bonding orbitals of the middle cyclobutane ring, while ^1^(π,π*) transitions of TOT and TOT-S are more delocalized over the wider π conjugation offered by the middle cyclobutadiene ring, as expected. This can also explain the consistent bathochromic shift observed for TOT and even more for TOT-S and the S_0_→S_1_ absorption band of this latter being centered at *ca.* 510 nm.

## 4 Conclusion

With this study, we have drawn the attention to an alternative design for efficient molecular solar thermal systems, not based on the most known and investigated norbornadiene/quadricyclane system, although maintaining the basic [2 + 2] photoisomerization principle to generate the high-energy isomer. Specifically, we have initially conceived a tricyclic diene that could photoproduce an all-carbon cubane, resulting in a highly strained structure that could improve both E_stored_ and D_storage_ values, compared to the norbornadiene/quadricyclane reference. Nevertheless, as also recently proposed by Li and Lopez (2020), the formation of a stable bicyclic triene is expected to severely compromise its potential use as a MOST system.

To overcome this problem, a modification of the low-energy isomer was conceived, consistently enlarging π conjugation by introducing a central cyclobutadiene moiety. This design solution offers the advantages of consistently red shifting the absorption spectrum until reaching the visible window and even decreasing its molecular weight, which is a beneficial issue for increasing the storage density. Moreover, depending on the photoproduced cubadiene (symmetric or distorted), the stored energy was found to be similar in its value or even increased with respect to the initially formulated system, hence resulting in a consistent improvement with respect to norbornadiene/quadricyclane.

The main drawback is nevertheless constituted by a decreased efficiency in the photochemical pathway, since the S_1_/S_0_ conical intersection leads to the photoproduct only if a fairly high ground-state energy barrier is overcome. Substitution effects on the proposed system were also taken into account by introducing a convenient *push*–*pull* effect that ideally changes the optical properties optimizing its absorption spectrum, which would cover the full visible window. Nevertheless, the same issue about a worsening photochemistry pathway can be raised and, moreover, coupled to a lower storage density.

All in all, we can conclude that photoisomerizing mechanisms leading to cubane or cubadiene derivatives could, in principle, constitute a valid alternative to the norbornadiene/quadricyclane system in terms of stored energy and storage density for improved MOST systems. Nevertheless, additional research, possibly coupling theoretical methods (Wang and Losantos, 2019) and experimental approaches, should be envisaged to maintain efficient photochemical pathways while avoiding the formation of stable by-products.

## Data Availability

The original contributions presented in the study are included in the article/Supplementary Material; further inquiries can be directed to the corresponding author.
