# Supplementary material for: Taking up the quest for novel molecular solar thermal systems: Pros and cons of storing energy with cubane and cubadiene
Source: Front Chem. 2023 Apr 12;11:1171848. doi: 10.3389/fchem.2023.1171848 (PMC10130657; doi:10.3389/fchem.2023.1171848)
Supplement: Supplementary file 1 [file DataSheet1.pdf]

Supplementary Material

Taking Up the Quest for Novel Molecular Solar-Thermal Systems: Pros and Cons of Storing Energy with Cubane and Cubadiene

Cecilia Merino-Robledillo[1], Marco Marazzi[1,2*]

[1]Universidad de Alcalá, Departamento de Química Analítica, Química Física e Ingeniería Química, Alcalá de Henares, Madrid, Spain.

[2]Universidad de Alcalá, Instituto de Investigación Química ‘‘Andrés M. del Río’’ (IQAR), Alcalá de Henares, Madrid, Spain.

*** Correspondence:**
Corresponding Author
marco.marazzi@uah.es

1 Supplementary Computational Details

The absorption spectra shown in Figure 8 of the main text were built as follows: by using the OpenMolcas software, each vertical transition from the electronic ground state is associated with a transition energy and a transition dipole oscillator strength (f_i). As an approximation to assign a certain shape to each transition, a simple method is the application of a Gaussian function to each transition, depending on an arbitrary Full Width at Half Maximum (FWHM), in this case 0.2 eV, as justified in the main text. Especially, the transition dipole strength for each i transition (D_i) can be related to the maximum molar absorptivity through the following equation:

$$D_i = 4\left[\frac{3 \cdot 1000 \cdot \ln(10) \cdot h \cdot c}{32 \cdot \pi \cdot N}\right] \varepsilon_i^{max} \sqrt{\pi} \frac{\sigma}{\tilde{\nu}_i} \tag{1.1}$$

where N is the Avogadro’s number, c is the speed of light, h is the Planck’s constant, σ is the standard deviation in wavenumbers, $\tilde{\nu}_i$ is the excitation energy in nm, and ε_i^{max} is the maximum molar absorptivity in $L\, mol^{-1} cm^{-1}$, corresponding to $\tilde{\nu} = \tilde{\nu}_i$ as incident electromagnetic radiation. On the other hand, D_i can be related to f_i by the equation:

$$f_i = \frac{8\pi^2 \tilde{\nu}_i m_e\, c}{3he^2} D_i \tag{1.2}$$

Finally, a convolution of the i Gaussians (each of them corresponding to photon absorption to an electronic excited state: $S_0 \rightarrow S_i$) is performed to sum up all vertical transitions in a resulting absorption spectrum:

$$\varepsilon_i(\tilde{\nu}) = \sum_{i=1}^{n} \varepsilon_i(\tilde{\nu}) = \sum_{i=1}^{n} \left(1.3062974 \times \frac{f_i}{\sigma} exp \left[- \left(\frac{\tilde{\nu} - \tilde{\nu}_i}{\sigma} \right)^2 \right] \right) \quad (1.3)$$

The whole procedure can be carried out by an in-house developed Python code.

2 Supplementary Figures

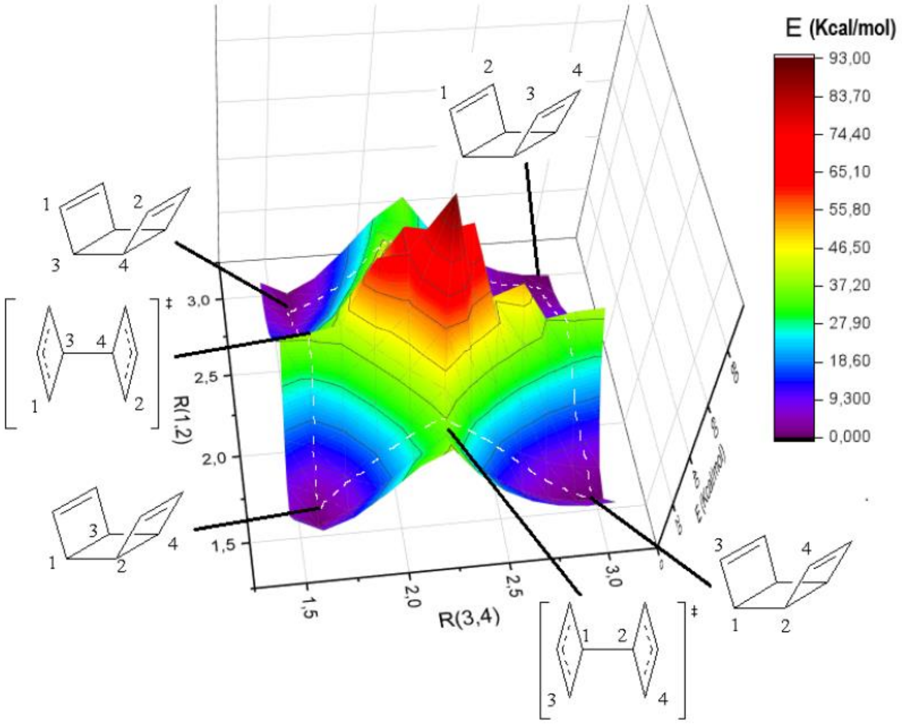

Supplementary Figure 1. CASSCF energy (E) as a function of C_1-C_2 and C_3-C_4 carbon-carbon distances (R(1,2) and R(3,4), in Ångström), resulting from a 2D-relaxed scan starting from the STOD optimized structure. As it can be seen, interconversion among the four chemically equivalent isomers of STOD can be obtained overcoming a barrier of *ca.* 30 kcal·mol^{-1} (dotted white line).

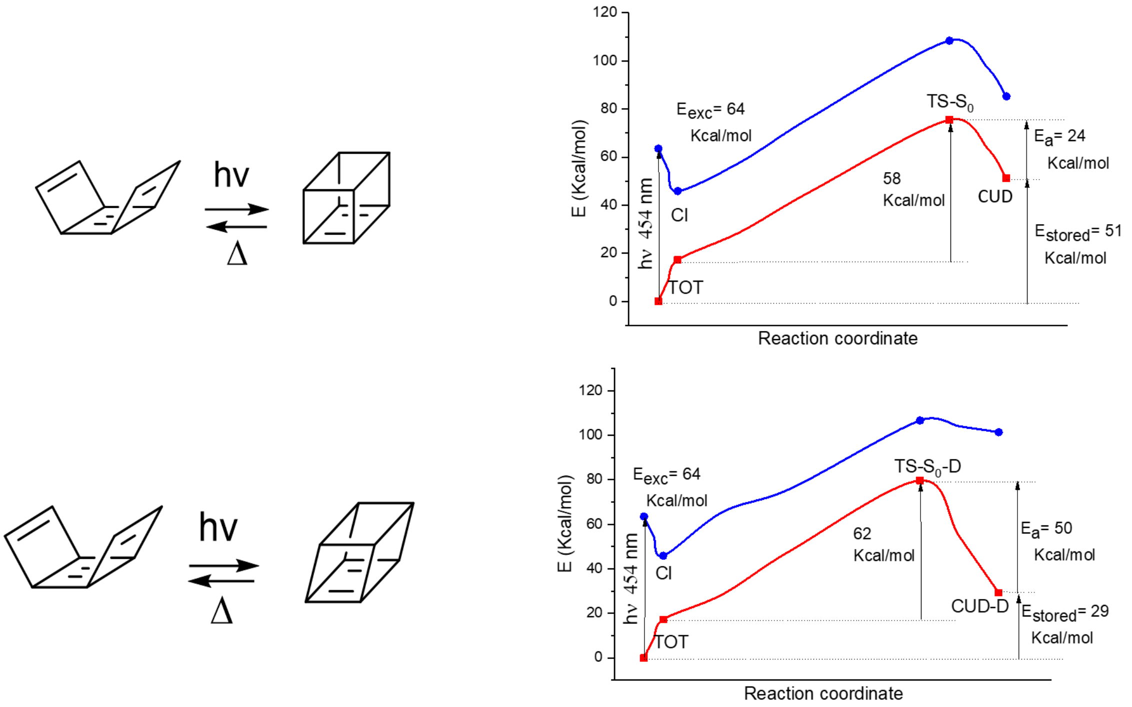

Supplementary Figure 2. CASPT2//CASSCF energy profiles for the formation of CUD and CUD-d by irradiating TOT.

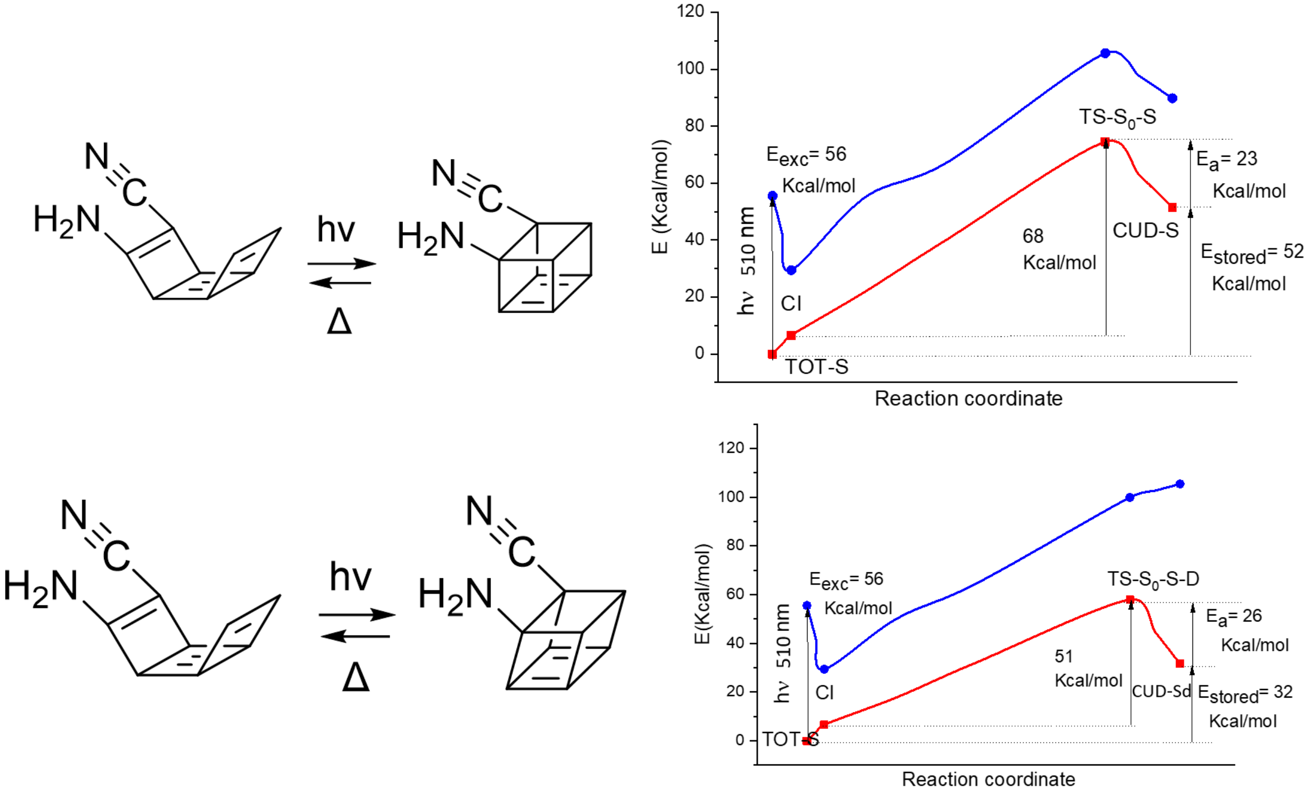

Supplementary Figure 3. CASPT2//CASSCF energy profiles for the formation of CUD-S and CUD-Sd by irradiating TOT-S. We note that the 2nd order perturbation has a much larger effect on E_{stored} of CUD-Sd, possibly due to the lower S_0 CASSCF reference weight (71% instead of *ca.* 90%).

3 Benchmark Calculations of the Absorption Spectrum of TOT-S

Supplementary Table 1. CASPT2 single point calculations of the TOT-S ground state minimum structure, including the differential energy, $\Delta(S_0\text{-}S_1)$, with respect to the level of theory employed in the work, CASPT2(8,8)/6-31G(d,p), and the wavefunction weight of the most representative configuration for both S_0 (closed shell) and S_1 (HOMO-LUMO) electronic states.

Level of theory	$\Delta(S_0\text{-}S_1)$ energy difference (kcal/mol)	CASPT2 S_0 main weight (%)	CASPT2 S_1 main weight (%)
CASPT2(8,8)/6-31G(d,p)	0	63.5	41.3
CASPT2(12,12)/6-31G(d,p)	-4.3	70.7	64.6
CASPT2(8,8)/ANO-S	-7.0	63.8	49.2
CASPT2(12,12)/ANO-S	-7.2	65.3	50.7
CASPT2(8,8)/ANO-L	-8.1	61.6	53.1
CASPT2(12,12)/ANO-L	-8.3	62.9	54.5

4 Cartesian Coordinates (in Ångström) of the Ground State Minima

ATOD

```
C        -0.840627    2.760574    2.861353
C        -0.120449    3.519142    2.008175
C        -0.833764    1.545617    1.966183
C         0.007276    2.431558    0.969741
C         0.403163    0.630467    2.196552
H        -1.781509    1.069071    1.697181
C         1.244198    1.516371    1.200073
H        -0.296610    2.633246   -0.062079
C         0.530890   -0.457162    1.158165
H         0.707065    0.428811    3.228374
C         1.251004    0.301397    0.304925
H         2.191953    1.992915    1.469042
H        -1.200307    2.909165    3.877338
H         0.320769    4.511357    2.075318
H         1.610653    0.152773   -0.711064
H         0.089672   -1.449381    1.091070
```

STOD

C	-1.11861	3.72304	3.34474
C	-0.66232	4.89141	2.83475
C	-1.00331	2.98683	2.03589
C	-0.47747	4.33375	1.44796
C	0.38885	2.30293	1.71408
H	-1.88045	2.48314	1.62032
C	0.9146	3.64989	1.12619
H	-0.96815	4.82002	0.60027
C	1.47765	2.44759	2.7446
H	0.33601	1.3943	1.10795
C	1.93359	3.61615	2.23474
H	1.24823	3.73125	0.08799
H	-1.3826	3.39945	4.34958
H	-0.42338	5.85646	3.27713
H	2.69369	4.32517	2.55668
H	1.73467	1.86803	3.62899

BCT

C	-1.150123	2.396263	2.782354
C	-0.784099	3.740162	2.661108
C	-0.373764	2.024219	1.514347
C	-0.032864	3.518036	1.503058
C	0.780966	1.046085	1.667210
H	-1.009883	1.715550	0.688567
C	1.567283	0.656899	0.361783
H	0.534701	4.138654	0.838246
C	0.446798	-0.444539	1.674156
H	1.429153	1.356391	2.482110
C	1.187623	-0.676462	0.510365
H	2.171811	1.196745	-0.340496
H	-1.759156	1.836323	3.464161
H	-1.003904	4.618397	3.239320
H	1.394915	-1.562984	-0.059989
H	-0.116077	-1.063829	2.344072

BOT

C	-0.48981	2.57432	2.98731
C	0.23197	3.33045	2.13492
C	-0.93213	1.58607	1.92364
C	-0.08352	2.47509	0.92146
C	-0.42999	0.19295	2.14315
H	-2.01059	1.59378	1.71361
C	1.11534	1.81188	0.31816
H	-0.69102	2.9762	0.15524
C	0.72335	-0.24787	1.59809
H	-1.01051	-0.45669	2.79696

C	1.50399	0.56995	0.67617
H	1.69926	2.38213	-0.4032
H	-0.67194	2.6123	4.05924
H	0.85636	4.21335	2.25439
H	2.40209	0.13852	0.23933
H	1.0705	-1.25649	1.8119

CUB

C	-0.506387	3.347860	3.332375
C	0.007021	4.704965	2.745115
C	-1.036257	2.936289	1.917998
C	-0.522859	4.293368	1.330749
C	0.344251	2.273659	1.593619
H	-2.013195	2.490508	1.715476
C	0.857649	3.630738	1.006370
H	-1.085915	4.941609	0.654809
C	0.874147	2.685217	3.007991
H	0.480224	1.293692	1.129594
C	1.387556	4.042322	2.420730
H	1.407504	3.744794	0.068930
H	-1.055713	3.234074	4.270143
H	-0.128610	5.684710	3.209678
H	2.364334	4.488121	2.623911
H	1.437230	2.037484	3.684374

TOT

C	-2.043000	0.815116	-1.595186
C	-2.112016	-0.544403	-0.939315
C	-0.658456	0.728862	-1.628196
C	-0.727479	-0.630721	-0.972421
C	-3.142651	0.024187	-0.028161
C	-3.079893	1.259502	-0.624053
C	0.470454	1.038330	-0.708703
C	0.407940	-0.197103	-0.113035
H	-3.592000	-0.313168	0.885375
H	-3.463594	2.218762	-0.336155
H	0.983242	1.941770	-0.442016
H	0.855074	-0.590435	0.778957

CUD

C	-2.151205	0.627222	-1.873618
C	-2.214882	-0.640101	-1.260098
C	-0.592790	0.527140	-1.916165
C	-0.657591	-0.741905	-1.301410

C	-2.148351	-0.042412	0.174255
C	-2.097721	1.381459	-0.517399
C	-0.486784	1.259340	-0.547318
C	-0.578246	-0.122301	0.128007
H	-2.733750	-0.358498	1.021709
H	-2.629456	2.283495	-0.264017
H	0.174608	2.076569	-0.312353
H	0.013786	-0.499309	0.945496

CUD-d

C	-4.448105	0.559435	-1.434971
C	-4.831331	-0.825584	-1.632451
C	-3.052172	0.747473	-1.727509
C	-3.389044	-0.676253	-1.645820
C	-3.035333	-0.934849	-0.193516
C	-2.869300	0.598594	-0.055804
C	-4.411386	0.660962	0.079600
C	-4.561021	-0.876563	0.061613
H	-4.988586	1.367087	0.647965
H	-5.141942	-1.491986	0.724723
H	-2.129675	1.135762	0.511099
H	-2.350395	-1.696369	0.131991

TOT-S

C	-4.562931	0.748158	-1.037269
C	-4.665768	-0.744054	-1.246052
C	-3.194862	0.707457	-1.268190
C	-3.293882	-0.787759	-1.429724
C	-2.046977	-0.886963	-0.624213
C	-1.970479	0.483953	-0.462369
C	-5.521830	0.594866	0.086497
C	-5.608419	-0.764529	-0.095737
H	-6.018495	-1.547591	0.511236
H	-5.843105	1.238597	0.881517
C	-1.418813	-1.933954	0.114873
N	-0.905749	-2.756326	0.710524
N	-1.169029	1.309380	0.256831
H	-1.515773	2.216007	0.467255
H	-0.592738	0.901786	0.958134

CUD-S

C	-4.595869	0.815029	-1.464676
C	-4.814528	-0.576020	-1.600497
C	-3.058036	0.600676	-1.654983
C	-3.282636	-0.783488	-1.789028

C	-3.117564	-1.042291	-0.261742
C	-2.877452	0.478422	-0.096112
C	-4.423535	0.700351	0.077983
C	-4.667754	-0.815053	-0.068629
H	-5.279661	-1.459593	0.538317
H	-4.825913	1.370927	0.818820
C	-2.373755	-2.120260	0.355851
N	-1.761737	-2.965368	0.860594
N	-1.946461	1.050565	0.789452
H	-1.990437	2.049939	0.780426
H	-1.008923	0.767514	0.585673

CUD-Sd

C	-4.599224	0.993227	-1.442949
C	-4.569165	-0.463091	-1.562324
C	-3.242190	0.523572	-1.541929
C	-3.217103	-0.894461	-1.873312
C	-3.120540	-1.075539	-0.235233
C	-2.936158	0.456031	-0.041330
C	-4.452738	0.709959	0.156940
C	-4.645534	-0.807851	-0.091619
H	-5.373520	-1.464154	0.347205
H	-4.910693	1.311135	0.922713
C	-2.352865	-2.111616	0.417014
N	-1.730632	-2.927926	0.955642
N	-1.912890	1.039540	0.720563
H	-1.957574	2.038342	0.712999
H	-1.003435	0.744192	0.427081
